# The Combined Effect of Caseinates, Native or Heat-Treated Whey Proteins, and Cryogel Formation on the Characteristics of Kefiran Films

**DOI:** 10.3390/molecules30153230

**Published:** 2025-08-01

**Authors:** Nikoletta Pouliou, Eirini Chrysovalantou Paraskevaidou, Athanasios Goulas, Stylianos Exarhopoulos, Georgia Dimitreli

**Affiliations:** 1 Department of Food Science and Technology, International Hellenic University, 57400 Thessaloniki, Greece; nikolettapouliou@gmail.com (N.P.); irinixp@gmail.com (E.C.P.); dimitrel@ihu.gr (G.D.); 2 Department of Hygiene and Technology of Food of Animal Origin, School of Veterinary Medicine, Aristotle University of Thessaloniki, 54124 Thessaloniki, Greece

**Keywords:** kefiran, sodium caseinates, whey protein concentrates, cryogels, glycerol, biodegradable films

## Abstract

Kefiran, the extracellular polysaccharide produced from the Generally Recognized as Safe (GRAS) bacteria in kefir grains, with its well-documented functional and health-promoting properties, constitutes a promising biopolymer with a variety of possible uses. Its compatibility with other biopolymers, such as milk proteins, and its ability to form standalone cryogels allow it to be utilized for the fabrication of films with improved properties for applications in the food and biomedical–pharmaceutical industries. In the present work, the properties of kefiran films were investigated in the presence of milk proteins (sodium caseinate, native and heat-treated whey proteins, and their mixtures), alongside glycerol (as a plasticizer) and cryo-treatment of the film-forming solution prior to drying. A total of 24 kefiran films were fabricated and studied for their physical (thickness, moisture content, water solubility, color parameters and vapor adsorption), mechanical (tensile strength and elongation at break), and optical properties. Milk proteins increased film thickness, solubility and tensile strength and reduced water vapor adsorption. The hygroscopic effect of glycerol was mitigated in the presence of milk proteins and/or the application of cryo-treatment. Glycerol was the most effective at reducing the films’ opacity. Heat treatment of whey proteins proved to be the most effective in increasing film tensile strength, reducing, at the same time, the elongation at break, while sodium caseinates in combination with cryo-treatment resulted in films with high tensile strength and the highest elongation at break. Cryo-treatment, carried out in the present study through freezing followed by gradual thawing of the film-forming solution, proved to be the most effective factor in decreasing film roughness. Based on our results, proper selection of the film-forming solution composition and its treatment prior to drying can result in kefiran–glycerol films with favorable properties for particular applications.

## 1. Introduction

Biodegradable polymers have become the focus of eco-friendly approaches, as opposed to non-degradable materials, for various applications in the food and pharmaceutical industries [1,2]. Among the most widely explored and promising alternatives for producing eco-friendly films are various natural polysaccharides, such as starch, chitosan, and fiber [3,4,5,6], and protein-based biopolymers (plant proteins, e.g., gluten, soy, and zein, and animal proteins, e.g., casein, whey protein, and gelatin) [7,8,9].

Kefiran, the water-soluble branched glucogalactan found in kefir and kefir grains [10] and biosynthesized by the metabolic activity of the generally recognized as safe (GRAS) lactic bacteria, has been widely recognized in the literature [2,11,12] for its health-promoting properties and its potential applications in the food, medicine, and nanotechnology fields [11,12]. Although difficult and expensive to produce compared to other similar ingredients, kefiran has many advantages, as it combines GRAS status with functional and health-promoting properties, and it has a broad range of applications. The applicability of kefiran is further enhanced by its unique ability to fabricate biodegradable films and cryogels upon freezing and thawing [12].

Kefiran films are naturally brittle due to the high concentration of hydroxyl groups in their structure. Plasticizers reduce intermolecular forces by weakening the hydrogen bonds of the polymer chains, thereby limiting the natural brittleness of polymer membranes. Glycerol is a small-sized plasticizer, with increased water-binding capacity, that can be introduced within polymer chains, increasing the moisture content of the matrix, reducing interactions between macromolecules, and resulting in increased flexibility. Thus, the incorporation of a plasticizer into kefiran films improves their mechanical properties and, as a result, expands their potential uses [13,14,15]. However, brittleness reduction and enhancement of elasticity with hygroscopic plasticizers such as glycerol are accompanied by a loss in the observed tensile strength of the formed membranes [16]. To this end, and in order to produce films with improved elongation and tensile properties, the presence of other biopolymers alongside kefiran and glycerol in the film-forming matrix could be investigated since their interactions could produce the desired outcome. Milk proteins, particularly whey proteins, and especially caseins, can be combined with kefiran as they represent highly promising, natural, and widely available biocompatible ingredients for the development of biodegradable films [17].

Caseins are the principal proteins in milk, making up to 75–80% of its total protein content [18,19]. They have a flexible structure with an open shape that can adapt to the surrounding environment and exhibit increased resistance to heat treatment [18]. Casein molecules encompass both hydrophilic and hydrophobic regions, which influence their functional properties [19]. Due to the abundance of electrostatic, hydrophobic, and intermolecular hydrogen bonds, caseins can form films from aqueous solutions. The most commonly used form of casein for this purpose is caseinates [18]. Several studies have explored the formation of films using caseins and caseinates, investigating their structural, mechanical, and functional properties [20,21,22,23,24,25,26,27]. In addition to casein, whey proteins, which represent around 17% of the milk’s proteinous content, are globular in shape, contain intramolecular disulfide bonds that stabilize their structure, and are easily susceptible to heat-induced denaturation [18]. Heat processing of whey proteins causes the macromolecules to unfold, exposing their disulfide bonds, which in turn prompts the formation of random sulfur bridges between all the proteins (whey or casein) found in a dairy system. More to the point, a complex is formed between beta-lactoglobulins and kappa-caseins [28,29,30], which strongly reflects the functionality of these molecules [28] and is expected to significantly affect the properties of kefiran membranes. Furthermore, due to their complex-forming ability, whey proteins have been reported to produce [18,31,32,33,34,35,36,37] stand-alone, transparent biodegradable membranes with good mechanical, physicochemical and barrier properties.

Several studies focused on the investigation of the film properties of kefiran either on its own or in mixtures with other biopolymers, such as starch [38,39], chitosan [40], sugars [41] or polyols [41,42]. Piermaria et al. [43] developed edible kefiran films with glycerol as a plasticizer that simultaneously served as carriers for probiotic microorganisms. The combined effect of kefiran and whey proteins alone [31] or together with titanium dioxide [32] on the film properties has also been reported. Nonetheless, to the best of our knowledge, there are no literature reports on the effect of casein salts on kefiran films or their combined effect with whey proteins.

Heat treatment is a processing step generally employed in cast-membrane production technology both for the dissolution of components and for sanitation purposes. Since the properties of milk proteins alter significantly when heat-treated—causing changes to their structure and intermolecular association—the application of heat treatment was considered necessary in order to examine whether it affects the characteristics of the membranes.

Furthermore, cryogel formation is the result of cryo-concentration of a polymer in solution that undergoes the process of freezing, subsequently followed by thawing. This topologically drastic increase in the polymer concentration, accompanying the formation of water crystals, promotes interactions within the polymer matrix, resulting in gel formation. When freezing, the solvent crystals gradually grow to the point that they meet the facets of other crystals, which after thawing create a system of interconnected pores in between the polymer chains, which withhold the water, creating a gel [44] and not a solution. This interesting property of kefiran has been studied [45,46,47] and it was proven that kefiran cryogels exhibited increased elasticity, due to their molecular interactions, making them a suitable material for several biomedical applications, such as scaffolds for delivery systems and tissue engineering applications [48]. Our previously published work demonstrated that cryogel formation, prior to the preparation of kefiran films, had a significant effect on membrane properties [49]. Molecular interactions of the polymer chains, as a consequence of the topologically increased concentrations, occurring during ice crystal formation, were the cause for increased film thickness and moisture content, reduced mechanical properties and smoother surface morphology.

In the present study, the properties of different biopolymers, capable of forming films, such as caseinates and whey proteins, were combined to create kefiran films with improved attributes, specifically aiming for a simultaneous increase in tensile strength and elasticity. Heat treatment and cryogel formation of the film-forming solutions were also employed, which, in combination with the milk proteins, was expected to lead to further enhancement of the membranes’ characteristics. Glycerol was used as a plasticizer.

## 2. Results

**Figure 1:**
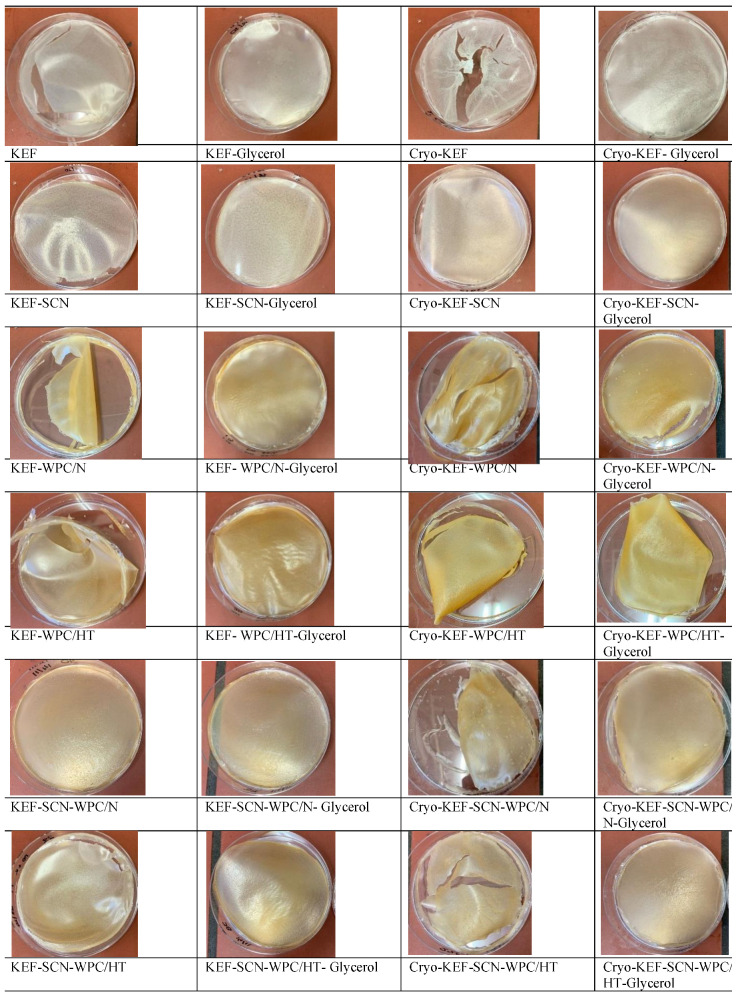
Images of kefiran films prepared with or without milk proteins (sodium caseinate—SCN, whey protein—WPC, native—N or heat-treated—HT), glycerol and cryo-treatment.

Figure 1 shows images of kefiran films prepared with or without milk proteins, glycerol and cryo-treatment. Sodium caseinates resulted in kefiran films with the finest and smoothest appearance, regardless of glycerol addition and cryo-treatment application. Whey proteins gave a yellowish color to the produced films that was credited to their riboflavin content [28]. Glycerol, irrespective of the application of cryo-treatment, improved film appearance, especially in the presence of whey proteins.

Cryo-treatment resulted in the formation of kefiran films (Cryo-KEF) with a crumbly appearance, which, in the presence of glycerol, changed to a smoother surface. The molecular interactions of the kefiran chains, due to their topologically increased concentrations occurring during freezing, is considered the reason for the crumbly appearance of the control kefiran films (Cryo-KEF), which, however, when glycerol was added to the film forming solution, became less intense, due to the hygroscopic nature of the specific ingredient, resulting in smoother surfaces [15]. The crumbliness caused by cryo-treatment was not evident in the presence of milk proteins, and the films appeared smooth without the need for glycerol addition. Whey proteins caused the local formation of cracks, but without affecting film smoothness.

Sodium caseinates produced kefiran films with a smooth appearance that became more transparent with increasing glycerol concentrations. According to our previously published results [49], the increment in glycerol concentration up to 120% resulted in the production of kefiran films with excellent transparency.

### 2.1. Structure of Cryogels

**Figure 2:**
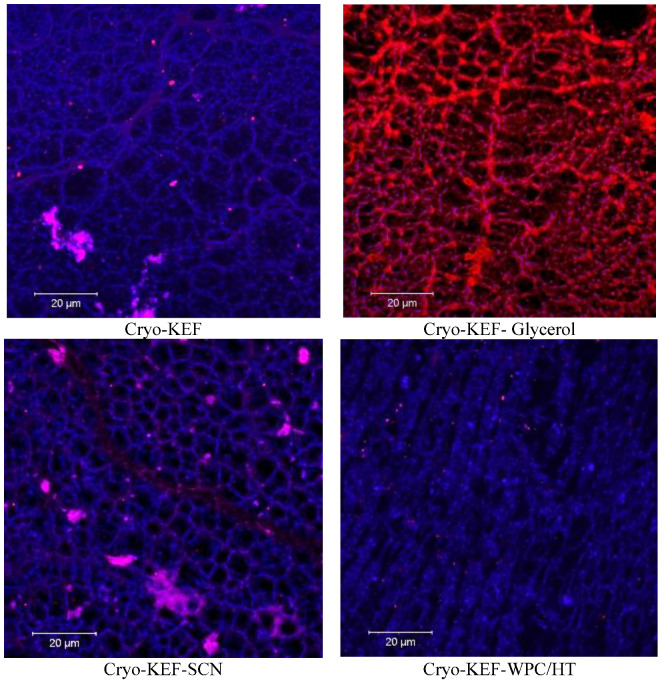
Surface morphology of kefiran cryogels prepared with or without milk proteins (sodium caseinate—SCN, whey protein—WPC, native—N or heat-treated—HT) and glycerol at magnifications of ×20.

As mentioned earlier, cryo-treatment, as implemented in the present work, involved freezing of the film-forming solution, which produced freeze-concentration phenomena, followed by thawing prior to drying to produce kefiran films. This freezing, thawing and drying process, apart from the locally increased concentrations of solids, which created a sponge-like (or honeycomb-like) net of filaments, also generated large void volumes created by the ice crystals during freezing. This matrix can be seen in the representative confocal microscope images presented in Figure 2. Further observation reveals that sodium caseinates retained the sponge-like appearance of the cryogel matrix, which, however, became more dense when whey proteins were added to the mixture.

### 2.2. Physical Properties of Films

#### 2.2.1. Thickness of the Kefiran Films

**Figure 3:**
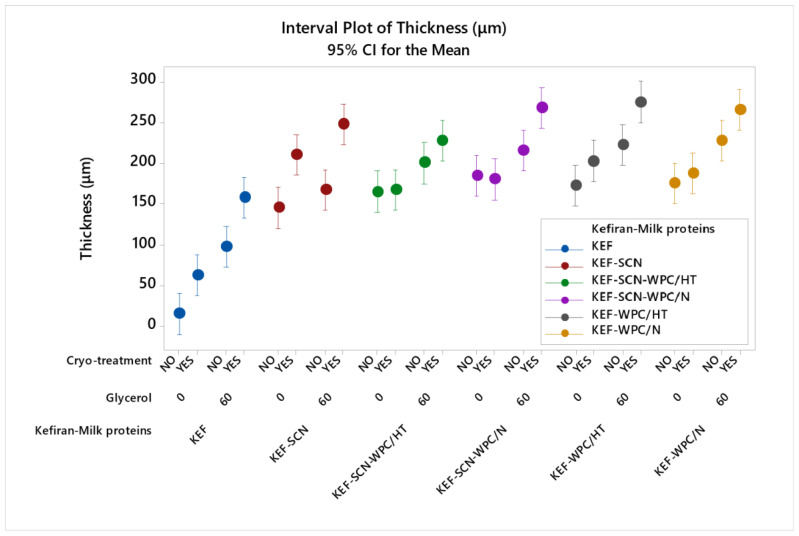
Thickness of kefiran films prepared with or without milk proteins (sodium caseinate—SCN, whey protein—, native—N or heat-treated—HT), glycerol and cryo-treatment. Vertical bars with no overlaps differ significantly.

Figure 3 shows the significant effect (*p* < 0.05) of the studied factors on film thickness. As can be seen, the thickness of kefiran films increased significantly in the presence of milk proteins. This increase was more pronounced in the case of sodium caseinates, when cryo-treatment was applied. In general, glycerol in combination with cryo-treatment significantly exhibited the highest values of thickness for kefiran–protein films. This combined effect of glycerol and cryo-treatment on kefiran films’ thickness was also evident in our previously published results [49]. Heat treatment of whey proteins did not significantly affect film thickness.

Increased film thickness, caused by the presence of milk proteins and glycerol, is the outcome of their hydrophilic nature and their ability to bind water molecules, leading to swelling and increasing the hydrodynamic volume of the polymer. This greater hydrodynamic volume of the macromolecules, in the film-forming solution, causes the formation of a less dense polymeric matrix, which, upon drying, results in films with greater thickness. Cryo-treatment of the film-forming solutions prior to drying further enhanced the experienced film thickness, due to the void volume created in the matrix by the formation of ice crystals, which, after drying, remained empty, reducing density but increasing thickness [49].

#### 2.2.2. Kefiran Film Moisture Content

**Figure 4:**
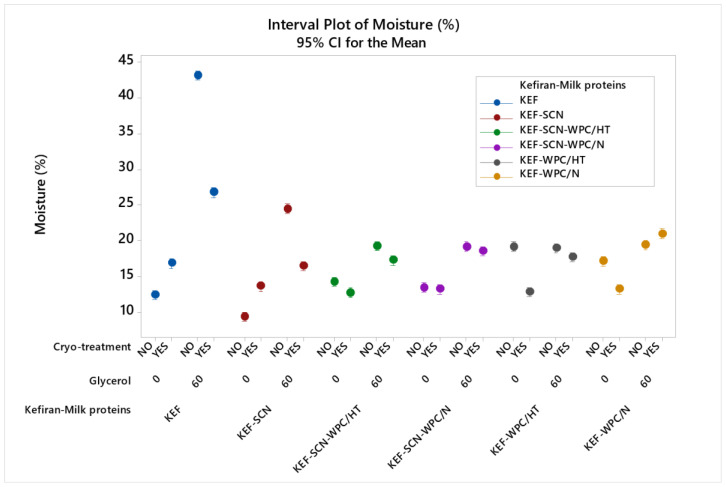
Moisture content of kefiran films prepared with or without milk proteins (sodium caseinate—SCN, whey protein—WPC, native—N or heat-treated—HT), glycerol and cryo-treatment. Vertical bars with no overlaps differ significantly.

Statistical analysis showed a significant effect (*p* < 0.05) on moisture content among the samples. According to Figure 4, moisture content of kefiran films without milk proteins was increased drastically by the presence of glycerol. The increase was also important, but lower, when sodium caseinates were used. Milk protein mixtures exhibited the lowest increment in their moisture content when glycerol was added. In general, milk proteins resulted in kefiran films with reduced moisture content, especially in the films where glycerol was added. Film moisture content was not affected by whey protein heat-induced denaturation.

Cryo-treatment increased the moisture content of the control sample (KEF) and that of the samples prepared with sodium caseinates, but it significantly reduced the moisture content of the corresponding films when these were prepared with glycerol. In contrast, in the presence of whey proteins, cryo-treatment did not increase film moisture, but in most cases, it rather reduced it when compared to the kefiran–whey protein films without cryo-treatment.

The increase in films’ moisture content caused by the presence of glycerol and its hydration effect [15] can be moderated by the application of cryo-treatment or the use of milk proteins and especially whey proteins. Particularly, the combined effect of cryo-treatment and whey proteins may result in films with comparable moisture values to the control sample (Figure 4). The reduction in the moisture content of the films that have been supplemented with glycerol in the presence of milk proteins can be attributed to the more dense structure of the films, which prevented moisture adsorption from the environment post-drying. Moisture content of films affects their flexibility, since water molecules interpose themselves and are retained among the polymer molecules, reducing the strength of their interactions and thus the films’ stiffness.

#### 2.2.3. Water Solubility of the Kefiran Films

**Figure 5:**
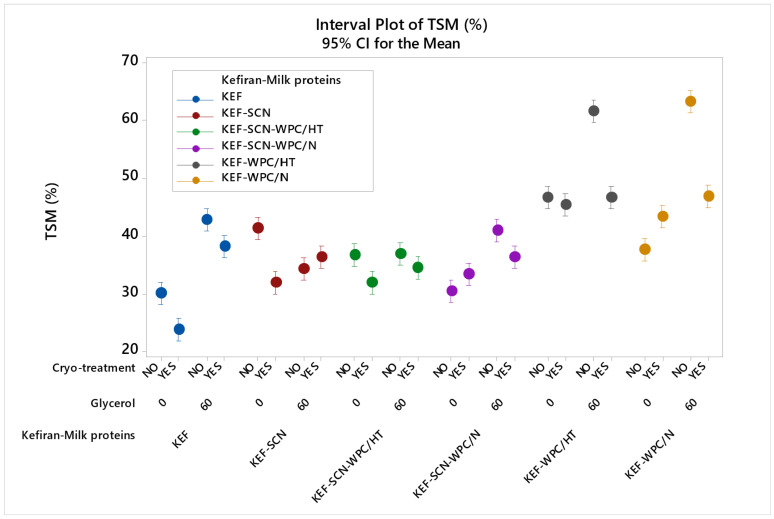
Total soluble matter (TSM) of kefiran films prepared with or without milk proteins (sodium caseinate—SCN, whey protein—WPC, native—N or heat-treated—HT), glycerol and cryo-treatment. Vertical bars with no overlaps differ significantly.

The effect of milk proteins, glycerol and cryo-treatment on the water solubility of kefiran films is presented in Figure 5. According to ANOVA, all factors studied exhibited a statistically significant effect (*p* < 0.05) on film solubility. As seen, films prepared with milk proteins exhibited increased values of solubility, with whey proteins (native or heat-treated) in the presence of glycerol showing the highest increment. The hydrophilic nature of whey proteins [28] and glycerol [16], which easily attract water molecules, is the cause for the increased water solubility experienced.

Cryo-treatment reduced water solubility of the control, KEF-SCN, KEF-SCN-WPC/HT and SCN-WPC/N–glycerol samples, with this effect being more pronounced on kefiran–whey protein films in the presence of glycerol. This effect of cryo-treatment on kefiran film solubility is in agreement with our previously published results [49]. The three-dimensional matrix created by the kefiran molecules and especially the dense polymer filaments created during cryo-treatment appear difficult to rehydrate and dissolve, and this effect becomes especially more profound in the presence of glycerol that is possibly trapped within the polymer chains, reducing its ability for water adsorption.

#### 2.2.4. Color Measurements of the Kefiran Films

**Figure 6:**
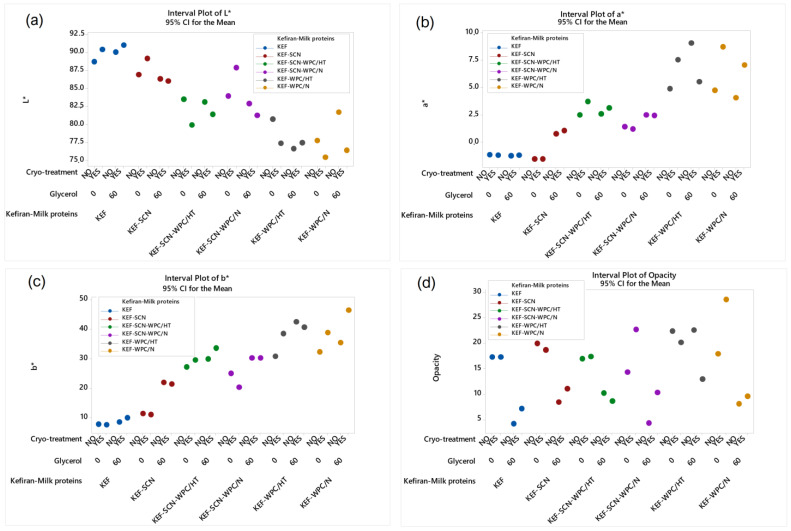
Color parameters (**a**): L*; (**b**): a*; (**c**): b* and opacity (**d**) of kefiran films prepared with or without milk proteins (sodium caseinate—SCN, whey protein—WPC, native—N or heat-treated—HT), glycerol and cryo-treatment. Vertical bars with no overlaps differ significantly.

According to ANOVA, all factors studied significantly affected (*p* < 0.05) the color parameters and opacity of the kefiran films, as shown in Figure 6. Whey proteins, native or heat-treated, reduced film brightness (Figure 6a), increasing the intensity of red (Figure 6b) and yellow (Figure 6c) color alongside opacity (Figure 6d). This is also evident from the images of kefiran–milk protein films presented in Figure 1. Caseinates, with or without the presence of native milk proteins, in the absence of glycerol, and combined with the application of cryo-treatment, resulted in maintaining film brightness. Kefiran film opacity greatly reduced in the presence of glycerol, irrespective of milk proteins and cryo-treatment. Glycerol also increased the films’ brightness only in the absence of milk proteins.

Color is an important parameter affecting film acceptance, and glycerol plays an especially significant role in reducing opacity, justifying its use in greater concentrations in order to improve film visual presentation (Figure 1). Further experimentation is required, with regards to film composition, in order to achieve the preparation of films with favorable color characteristics. Caseinates did not negatively affect film color. In fact, in combination with increased glycerol concentrations is believed to achieve films with favorable color and physical appearance.

### 2.3. Water Adsorption Isotherms

**Figure 7:**
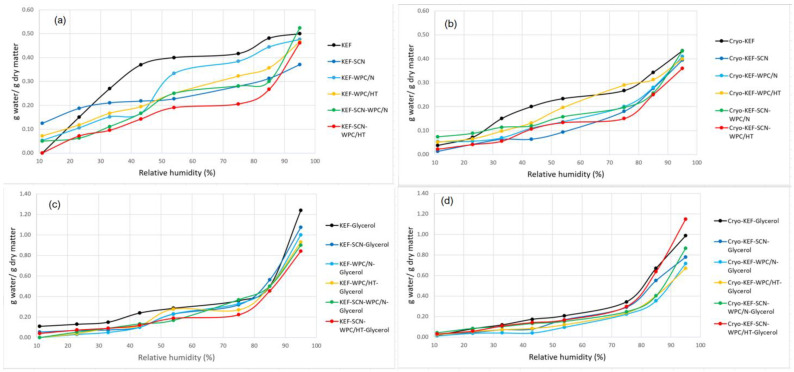
Adsorption isotherms of kefiran films prepared with or without milk proteins (sodium caseinate—SCN, whey protein—WPC, native—N or heat-treated—HT), glycerol and cryo-treatment. Kefiran–milk protein films without glycerol and cryo-treatment (**a**); kefiran–milk protein films with cryo-treatment application and without glycerol addition (**b**); kefiran–milk protein films with glycerol addition and without cryo-treatment (**c**); kefiran–milk protein films with glycerol and cryo-treatment (**d**).

Adsorption isotherms of kefiran films are shown in Figure 7. As can be observed in Figure 7a, milk protein incorporation in the films resulted in reducing water vapor adsorption capacity, especially at higher relative humidity values. In the presence of sodium caseinates and especially in their mixture with heat-treated whey protein concentrates, this effect was more pronounced. The application of cryo-treatment, without glycerol addition (Figure 7b), caused a slight decrease in water vapor adsorption capacity for all kefiran films, something that was evident, to a higher degree, in the control films with native whey proteins. Glycerol addition caused an increase in water absorption for all samples (Figure 7c,d). However, this effect was not so pronounced, especially at the highest relative humidity, when cryo-treatment was applied (Figure 7d). These results of water adsorption capacity are in good agreement with the experienced moisture contents of the films produced, since much of the moisture content measured is the outcome of environmental humidity adsorption.

Water vapor adsorption capacity is an important film property since it determines films’ permeability to moisture. Hygroscopic glycerol molecules inserted between polymeric chains decrease intermolecular forces, resulting in increasing molecular mobility in the film matrix, creating greater free volume and segmental motions, which facilitate the migration of water vapor through films [16] and increase water vapor adsorption. The reduced water vapor adsorption in the presence of milk proteins can be attributed to the more dense matrix created in their presence.

### 2.4. Kefiran Films’ Mechanical Properties

**Figure 8:**
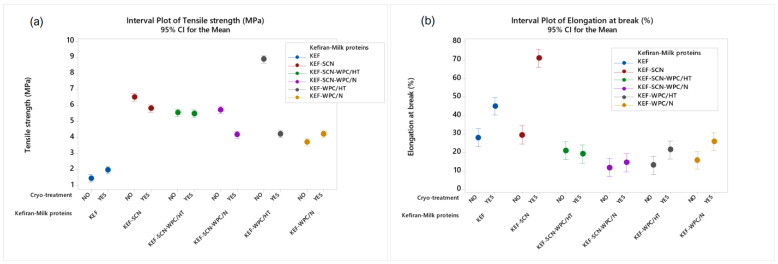
Mechanical properties (**a**): tensile strength; (**b**): elongation at break of kefiran films prepared with or without milk proteins (sodium caseinate—SCN, whey protein—WPC, native—N or heat-treated—HT), glycerol and cryo-treatment. Vertical bars with no overlaps differ significantly.

According to ANOVA, all factors studied exhibited a significant effect (*p* < 0.05) on films’ mechanical properties. As can be observed in Figure 8, kefiran films prepared with the incorporation of milk proteins exhibited higher values of tensile strength when compared to the control samples (without proteins). Heat-treated whey proteins, without cryo-treatment, exhibited the highest tensile strength. This increase in tensile strength in the presence of milk proteins can be credited to the increased concentration of macromolecules in the system, which form cross-linkages between them, creating a more dense and durable structure with regards to tensile strength. This effect is further enhanced through heat-induced denaturation of the milk proteins, which further increases the formation of cross-linkages between them.

Cryo-treatment slightly increased the tensile strength of the control samples and the films with native whey proteins, while it reduced tensile durability of all the other samples besides KEF-SCN-WPC/HT, which showed no statistical difference. The most intense decrease in the films’ tensile strength by the application of cryo-treatment was observed in the case of heat-treated whey proteins. Generally, cryo-treatment of the filmogenic solution prior to drying results in a more loose and swollen grid within the film structure, which negatively affects the mechanical strength of the films, since their thickness, which is increased by cryo-treatment, is taken in account in the calculation of tensile strength.

As concerns elongation at break, sodium caseinates in combination with cryo-treatment resulted in the formation of films with the highest elasticity. In general, kefiran films in the presence of milk proteins exhibited low values of elasticity but cryo-treatment improved the elongation at break values observed. Generally, as previously mentioned, the presence of caseins and heat-denatured whey proteins increases the number of intra- and inter-molecular bonds, enhancing the tensile endurance of matrices but at the same time reducing their elasticity. Contrary to the aforementioned, with regards to the effect of the proteins, the looser structures generated by cryo-treatment exhibit greater elasticity.

### 2.5. Structural Morphology of Kefiran Films

**Figure 9:**
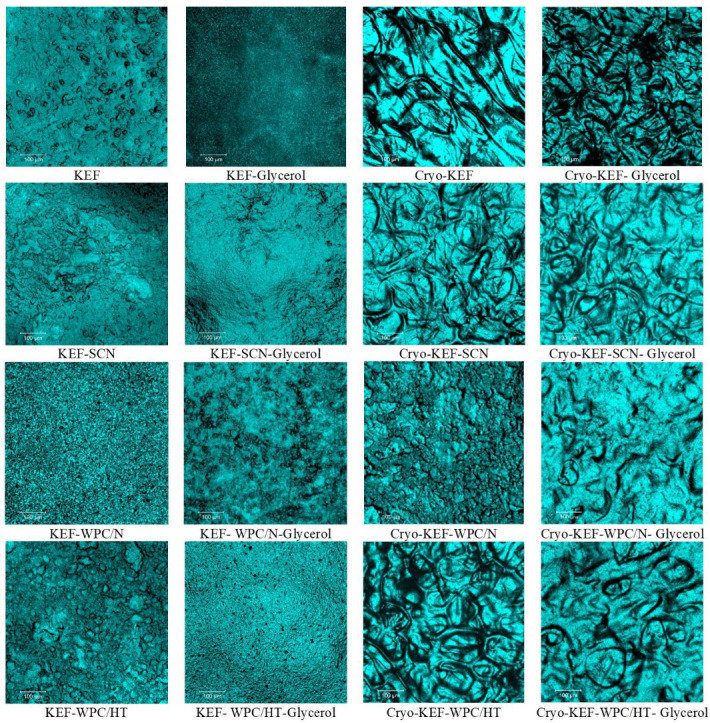
Surface morphology of kefiran films prepared with or without milk proteins (sodium caseinate—SCN, whey protein—WPC, native—N or heat-treated—HT), glycerol and cryo-treatment at magnifications of ×100.

**Figure 10:**
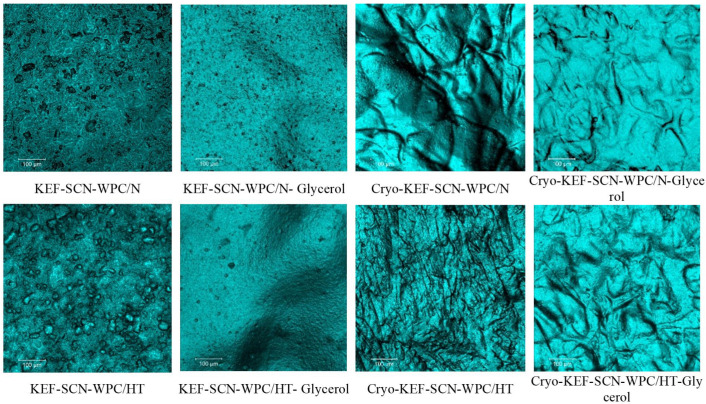
Surface morphology of kefiran films prepared with or without milk protein mixtures (sodium caseinate—SCN, whey protein—WPC, native—N or heat-treated—HT), glycerol and cryo-treatment (Cryo) at magnifications of ×100.

Figure 9 and Figure 10 show the confocal microscope micrographs of kefiran films with milk proteins (sodium caseinates and whey proteins native or heat-treated), their mixtures, respectively, with or without glycerol and the application of cryo-treatment. Kefiran films without glycerol and cryo-treatment appear to have a very rough surface, which in the case of native whey proteins is more compact. Addition of glycerol resulted in reducing film roughness, but cryo-treatment was the most effective in improving film smoothness. Again, native whey proteins presented the least smooth surface, a characteristic that is evident only when they are incorporated alone into the film’s matrix. Heat-denaturation of whey proteins in combination with glycerol and cryo-treatment reduced film roughness.

The increased smoothness of kefiran films, subjected to cryo-treatment, can be attributed to the macromolecules’ alignment that occurs during the freezing process. The effect of this alignment is even more evident in the case of heat treatment where protein aggregates of increased size are formed. Moreover, in the presence of glycerol, a less compact and more uniform structure is achieved due to its water holding and consequently swelling capacity. Cryo-treatment followed by glycerol is the most important parameter affecting film smoothness.

## 3. Discussion

### 3.1. The Effect of Glycerol on Film Properties

The hydrophilic nature of glycerol and its ability to intervene between kefiran molecules weakens their intermolecular interactions, increasing kefiran film thickness, moisture content, solubility, water vapor adsorption, elongation at break, surface smoothness, and reducing opacity and tensile strength. It has been reported that plasticizers destabilize interpolymeric hydrogen bonds, reducing intermolecular forces, and thus increasing the mobility and the space between polymer chains. As a consequence, they reduce tensile strength, while increasing the elongation at break and the flexibility of the polymer matrix. In addition, plasticizers usually increase the hydration of the film and decrease the water vapor barrier properties [15]. The hydration effect of glycerol was evident at the FTIR spectra of kefiran films [41,50]. These effects were in agreement with the findings of the present study, which have also been reported by Ghasemlou et al. [16]. In contrast, Piermaria et al. [41] reported that glycerol did not affect kefiran films’ thickness, enhancing the experienced tensile strength, reducing elongation at break and opacity.

### 3.2. Effect of Cryo-Treatment on Film Properties

As already described in our previously published work [49], during the freezing and thawing cryo-treatment of kefiran solutions, the hydrophilic nature of kefiran and the intramolecular interactions of the polysaccharide chains that arise because of their topologically increased concentrations occurring due to freeze concentration phenomena [12] are the main causes of the changes observed to the fabricated kefiran films. Cryo-treatment creates filament structures of kefiran molecules, as they occur after drying of the cryogel and create a sponge-like film matrix, resulting in increased film thickness. Some water molecules attached to polysaccharide molecules are trapped in the filaments, creating the three-dimensional structure of the kefiran cryοgel matrix, whereas their majority migrate and crystalize as ice. Part of this trapped water is possibly maintained in the film matrix after drying, resulting in increased moisture contents. The structure of the three-dimensional matrix of kefiran, formed during cryo-treatment, causes an increase in both tensile strength and elongation at break of kefiran–glycerol films and does not allow mobility of water molecules within the polymer chains, resulting in this way in decreased water solubility and water vapor adsorption. Moreover, macromolecule alignment, arising during cryogel formation, alongside matrix hydration are considered as the main the causes for the improved smoothness of the films’ surfaces.

The plasticizing–hydration effect of glycerol was moderated when cryo-treatment was applied, which resulted in reducing the kefiran–glycerol film moisture, water solubility and adsorption. This was attributed to the entrapment of glycerol within the chains of kefiran, as freeze concentration occurred, which subsequently after drying could not be rehydrated effectively via the absorption of environmental humidity. This hypothesis needs to be further confirmed with more analytical techniques, such as FTIR spectroscopy, Thermogravimetric Analysis (TGA) and Differential Scanning Calorimetry (DSC).

It is worth mentioning that the thickness of the control sample (KEF) was increased further with the combined effect of cryo-treatment and glycerol. The ability of glycerol to infringe between kefiran molecules and weaken their intermolecular interactions results in the formation of a three-dimensional matrix with more open structure during cryo-treatment. This matrix allows water molecules to be drawn away during the drying process; however, due to the increased intramolecular interactions of the polysaccharide chains, it maintains its structure, causing the films’ thickness to increase.

### 3.3. The Effect of Milk Proteins on Film Properties

Changes observed in kefiran–milk protein films can be attributed to the hydrophilic nature of both macromolecules being present. Whey proteins (native) are globular water-soluble molecules found in milk serum, while caseins are flexible molecules that possess both hydrophilic and hydrophobic interactions and are found in milk in the form of casein micelles. Casein micelles have their hydrophilic bonds exposed to their surface, staying this way in colloidal dispersion in the aqueous phase of milk. Globular whey proteins locate their hydrophobic side groups in the core of their folded molecule, alongside hydrophilic ones. Heat treatment of whey proteins causes unfolding of their molecules, which exposes both hydrophilic and hydrophobic side groups. Hydrophobic side groups subsequently form intermolecular hydrophobic interactions, while hydrophilic groups increase the molecules’ water holding capacity [28].

The increase in moisture content and solubility of the kefiran–milk protein films can be attributed to their hydrophilicity. However, in the presence of glycerol and/or the application of cryo-treatment, competition of milk proteins and glycerol for attaching water molecules caused a reduction in the moisture content and the water intake (reduced solubility and water absorption). Our hypothesis about competition for attaching water molecules can be supported by the findings of Gagliarini et al. [31], who reported that the FTIR spectroscopy revealed that whey proteins in the presence of kefiran favored water release, thus leading to a lower water content in the films.

With regards to color of the kefiran–milk protein films, it was greatly affected by the yellowish color of the whey protein concentrates, and further experiments are needed to find a whey protein preparation that will not negatively affect the color parameters.

The increase in tensile strength of the kefiran films in the presence of milk proteins can be attributed to the increased interactions into the kefiran–milk protein system. The interactions arising during heat denaturation of whey proteins resulted in the production of films with the highest tensile strength. Besides heat-treated whey proteins, sodium caseinates also proved to be effective in increasing film tensile strength. As expected, the elongation at break of these films reduced for the same reasons discussed previously. In the case, however, of sodium caseinates, the application of cryo-treatment resulted in films with the highest elongation, which could be explained by the greater flexibility of the sponge-like matrix experienced in these samples.

As concerns the effect of milk proteins on structure of the films, heat denaturation of whey proteins in combination with glycerol and cryo-treatment reduced the observed roughness.

## 4. Materials and Methods

### 4.1. Materials

For the preparation of the samples, kefiran with a moisture content of 4.54% (*w*/*w*) [49] was used, along with sodium caseinates (SCN-MIPRODAN 30, Arla Food Ingredients, Viby, Denmark), whey protein concentrate (WPC–Hellenic Protein S.A., Athens, Greece), and the plasticizer glycerol (Sigma-Aldrich Corp., St. Louis, MO, USA). Additionally, the salts LiCl, CH_3_COOK, MgCl_2_, K_2_CO_3_, Mg(NO_3_)_2_, NaCl, KCl, and KNO_3_ (Merck KGaA, Darmstadt, Germany) were used.

Kefiran isolation from kefir grains and the purification methodology followed have been reported elsewhere [49,51]. Briefly, proliferation of kefir grains was achieved by consecutive cultivations in batch laboratory and pilot-plant fermentations using skimmed ultra-high-temperature (UHT) milk. Fermentation conditions were performed with 1.5% (*w*/*w*) of kefir grains in the milk, at a temperature of 25 °C, under constant mild agitation, with no aeration of the culture and down to a final pH value of 4.5. Subsequent to the kefir grains’ mass increase, the polysaccharide was detached from the bacterial cells by heating at 80 °C in distilled water under constant vigorous agitation. Bacterial cells and proteins were removed via centrifugation, following the treatment of the crude kefiran solution with tricloroacetic acid (Merck KGaA, Darmstadt, Germany), or hypercloric acid (Carlo Erba Reagents SAS, Val de Reuil, France). Both acids exhibited the same efficacy in protein removal (both kefiran preparations had the same protein concentration) but hypercloric acid was preferably used at large scale, due to its lower cost. At least three consecutive ethanol or acetone precipitation steps, separated by intermediate dissolutions in distilled water, were applied to the supernatant liquid that contained the polysaccharide to purify it from the low-molecular-weight contaminants.

Chemical analysis (moisture, total nitrogen and total sugar content) [52] and the H-NMR spectrum of the resulting freeze-dried kefiran preparation were performed to confirm its purity and identity [49].

### 4.2. Experimental Design

For the preparation of the films, kefiran was added at a concentration of 3% (*w*/*w*) of the total film-forming solution, which, based on our previously published work [49], results in films with adequate properties. Milk proteins (sodium caseinates, whey proteins and a mixture of them at a ratio of 1:1) were chosen to be used at the same concentration as kefiran (3%, *w*/*w*). Since whey proteins are susceptible to heat denaturation, which results in changes in their structure and functionality, they were studied in both native and denatured states (by the application of heat treatment). Glycerol, added at 60% (*w*/*w*) calculated on the basis of dry kefiran, was used as a plasticizer, since it proved to be more effective, when compared to sorbitol, in improving film properties [49]. According to preliminary experiments, the concentration of glycerol, at 60% (*w*/*w*), proved effective in the production of films with good physicochemical and mechanical properties for all constituents used (kefiran and milk proteins).

A total of 24 film samples were prepared with or without cryo-treatment application. Particularly, a control kefiran film without proteins and glycerol addition was prepared (KEF), alongside 5 kefiran films without glycerol but with the addition of milk protein sodium caseinate (KEF-SCN), native whey protein (KEF-WPC/N), heat-treated whey protein (KEF-WPC/HT), and two mixtures of sodium caseinates and whey proteins at a ratio of 1:1 (sodium caseinates and native whey protein concentrates: KEF-SCN-WPC/N; sodium caseinates and heat-treated whey protein: KEF-SCN-WPC/HT) (Table 1). All the above-mentioned samples were also prepared with the addition of glycerol (a total of 12 samples). A second series of 12 kefiran films was fabricated with or without the application of cryo-treatment (a total of 24 film samples).

### 4.3. Preparation of the Kefiran Films

Kefiran solutions: The polysaccharide was dissolved in the required quantity of water by heating under continuous agitation at 80 °C, followed by the addition of glycerol, where it was necessary. Kefiran solutions were subsequently transferred to Petri dishes.

Kefiran–milk protein solutions: Milk proteins (sodium caseinates, native whey proteins, and mixtures of them at a ratio of 1:1) were dissolved in half of the required quantity of water under continuous stirring and then were added to the kefiran solution, prepared as described above with the other half of the required quantity of water. In the case of denatured whey proteins (heat-treated whey proteins, mixture of sodium caseinates and heat-treated whey proteins at a ratio of 1:1), the solution was heated-treated at 80 °C for 15 min before its addition to the kefiran solution. The glycerol was then added, according to the respective recipe; the solutions were then mixed well and subsequently transferred to Petri dishes.

Cryo-treatment: For the application of cryo-treatment, the kefiran or kefiran–milk protein solutions were frozen at −18 °C for 24 h, followed by thawing at 4 °C for 24 h.

Films fabrication: Film-forming solutions or cryogels were dried to a constant weight in a laminar air flow oven at 40 °C. Following drying, the films were stored in a controlled environment at 25 °C with 55% relative humidity for at least 2 days before further analysis.

### 4.4. Determination of the Physical Properties

The prepared kefiran films were initially photographed, and any surface irregularities were recorded.

#### 4.4.1. Thickness

The thickness of the films was measured in millimeters using a digital caliper with a resolution of two decimal digits and an accuracy of 0.1 mm. Six thickness measurements were taken for each film, covering both internal and peripheral points. The average thickness value for each sample was derived from these measurements.

#### 4.4.2. Moisture Content

The gravimetric standard method was used for moisture content determination by drying film samples at 102 ± 1 °C to constant weight [52].

#### 4.4.3. Water Solubility

The determination of water solubility was carried out as described by Exarhopoulos et al. [49]. Specifically, porcelain crucibles were pre-dried at 102 ± 1 °C and then placed in a desiccator with silica gel for 15 min prior to weighing, in order to stabilize their temperature. A film sample of 1 × 1 cm was then placed in each pre-weighed crucible, the weight was recorded, and the samples were dehydrated in an oven at 50 ± 1 °C for 24 h. The next day, the crucibles were weighed using an analytical balance to determine the dry weight of the films.

To complete the water solubility test, the pre-weighed film pieces were immersed in 50 mL of distilled water and maintained at 25 °C for 6 h under mild periodic agitation. After the 6 h period, the swollen film pieces were weighed and subsequently dried at 102 ± 1 °C for 24 h. At the end of the drying process, the final dry weight of the films after dehydration was recorded. Based on these measurements, the Total Soluble Matter (TSM%) of the films was calculated using the following equation:

TSM% = (A − B)/A × 100

where:

A refers to the initial dry weight of the films;
B refers to the final dry weight of the films.
Water solubility tests were conducted in duplicate.

#### 4.4.4. Color

Color was assessed using the CIELab scale, and the evaluated color parameters included L* (brightness), a* (+red color to –green component), b* (+yellow to –blue component), WI-ASTM (whiteness index) and opacity. For the measurements a non-contact imaging spectrophotometer (MetaVue VS3200, X-Rite, Inc., Grand Rapids, MI, USA), with the lighting condition set to daylight D65 and the measurement method defined as OLOD (Overlight Overdark) were used. In the case of film opacity, the opacity index was calculated by measuring the contrast of a film sample placed first on a calibrated white plate and then on a black calibration plate. The color standard of the white plate (L* = 94.8, a* = −0.78, and b* = 1.43) was used as the background reference for instrument calibration. In order to complete the measurement, a piece of film was first placed on the white side of the calibration surface, followed by measurement on the black side. All color measurements were carried out using the colorimeter in triplicate.

### 4.5. Film Vapor Adsorption Isotherms

Three pieces were cut from each film sample, each measuring 1 × 1 cm^2^, and were placed in pre-weighed and pre-dried plastic caps. The samples were then dried at 50 ± 1 °C for 24 h and subsequently weighed using an analytical balance. The dried film pieces were then placed above supersaturated salt solutions of varying relative humidity (RH) levels. These salt solutions were contained in glass vessels, which were hermetically sealed after placing the film samples inside. The RH values tested were as follows: LiCl (11%), CH_3_COOK (23%), MgCl_2_ (33%), K_2_CO_3_ (43.2%), Mg(NO_3_)_2_ (54%), NaCl (75%), KCl (85%), and KNO_3_ (95%). The vessels were stored at 20 °C for 7 days since preliminary tests showed that equilibrium was achieved within this period. At the end of this period, the film pieces were weighed again to calculate the water vapor adsorption for each sample. Moisture adsorption was plotted in a diagram showing grams of water per gram of dry matter versus relative humidity. The grams of water correspond to the difference in sample weight before and after equilibrium was reached, while the grams of dry matter refer to the initial dry weight of each sample. In this way, adsorption curves were generated for each sample, with relative humidity values of the salts on the x-axis and moisture adsorption on the y-axis.

### 4.6. Mechanical Properties of the Kefiran Films

The mechanical properties of the films in the presence of glycerol (films without glycerol addition were too brittle to be measured) were evaluated at ambient temperature (25 °C) using a texture analyzer (TA-XT Plus Universal Texture Analyzer, Stable Microsystems, Surrey, UK), following the basic ASTM D882 method [53]. Each film was initially cut into at least six strips, each measuring 60 mm in length (including 20 mm for gripping—10 mm on each side) and 9 mm in width. Specifically, using the A/TG tensile grip, each specimen was mounted on the instrument and stretched at a speed of 50 mm/min. The test was performed in triplicate for each sample. Stress–strain curves were recorded during the tensile test, from which the following properties were determined: tensile strength (Stress, MPa) and elongation at break (%). The data was extracted and processed using the TE32 software (v.6.1.7.0).

### 4.7. Surface Morphology of the Kefiran Films and Cryogels

The film surface morphology was examined using a confocal laser scanning microscope (CLSM Carl Zeiss LSM 700, Carl Zeiss Microscopy GmbH, Jena, Germany). The films were placed with double-sided tape on glass microscope slides and their surface was observed. The film observation was performed using a 405 nm laser. The objective lenses used were ×10 and ×20 magnifications. To monitor films with surface irregularities, the Z-Stack function of the microscope was used, which enables multilayer imaging at different focal depths. In this way, a comprehensive three-dimensional representation of the film surface was obtained.

The matrix of the cryogels produced during the freezing–thawing process of the film-forming solutions was also observed by the use of the confocal laser scanning microscope. A small amount of the film-forming solutions was transferred into the glass microscope slides, one drop of a Rhodamine B solution at 1% concentration was added and cover slips were placed on the surface of the samples. Then the samples were frozen at −18 °C for 24 h, followed by defrosting at 4 °C for 24 h. An oil immersion objective lens was used at ×63 magnification.

### 4.8. Statistical Analysis of the Experimental Data

Analysis of Variance (ANOVA) was applied to experimental data by the use of Minitab 18 statistical software. Particularly, for the physicochemical properties, three-way ANOVA was used to study the effect of milk proteins, glycerol and cryo-treatment. In the case of mechanical properties, two-way ANOVA was applied, since kefiran films without glycerol addition were too brittle to be measured. The two factors studied were milk protein addition and cryo-treatment application. The results are displayed as the mean values of measurements with 95% confidence intervals based on the pooled standard deviation of the Analysis of Variance.

## 5. Conclusions

Integration of milk proteins into kefiran–glycerol films can further improve their properties and, in combination with cryo-treatment, can result in membranes with different characteristics and thus possible applications. Cryogel formation prior to kefiran films fabrication especially affected film thickness and smoothness. The presence of glycerol in the film-forming solutions increased elasticity, moisture content, water solubility and vapor adsorption, but its effect was downsized by cryo-treatment and/or the incorporation of milk proteins in the matrix. Glycerol was the most effective parameter in reducing film opacity irrespective of the presence of milk proteins and cryo-treatment. Protein heat treatment did not significantly affect the physical properties of the films, but its effect was significant on their mechanical attributes. In general, milk proteins resulted in the fabrication of kefiran films with increased tensile strength and reduced elasticity when compared to control samples (without proteins). Moreover, sodium caseinates in combination with cryo-treatment resulted in films with good tensile strength and the highest elongation at break.

## Figures and Tables

**Table 1:** Film formulation and sample coding *.

Kefiran	Milk Proteins		Coding
	Type	Heat Treatment	
YES	NO	-	KEF
YES	Sodium caseinates	NO	KEF-SCN
YES	Whey proteins	NO	KEF-WPC/N
YES	Whey proteins	YES	KEF-WPC/HT
YES	Sodium caseinates: Whey proteins	NO	KEF-SCN-WPC/N
YES	Sodium caseinates: Whey proteins	YES	KEF-SCN-WPC/HT

* These formulations were repeated with or without glycerol (Glycerol) addition and the application of cryo-treatment (Cryo).

## Data Availability

The raw data supporting the conclusions of this article will be made available by the authors on request.
